# Systematic large-scale meta-analysis identifies miRNA-429/200a/b and miRNA-141/200c clusters as biomarkers for necrotizing enterocolitis in newborn

**DOI:** 10.1042/BSR20191503

**Published:** 2019-09-24

**Authors:** Hong Liu, Yi-Biao Wang

**Affiliations:** 1 Department of Pediatrics, Linyi People's Hospital, Cheeloo College of Medicine, Shandong University, Linyi 276000, P.R. China; 2 Department of Pediatrics, The Second Hospital of Shandong University, Jinan 250033, P.R. China

**Keywords:** Bioinformatics analysis, Differentially expressed genes, Differentially expressed microRNAs, Necrotizing enterocolitis in newborn

## Abstract

Necrotizing enterocolitis (NEC) is a critical neonatal disease with a high mortality. The possibility that miRNAs may play an important role in NEC has raised great attention. Hence, the present study identified biomarkers that affected NEC in newborn progression through miRNA and gene expression profile analysis. miRNA chip GSE68054 and gene chip GSE46619 of NEC in newborn were analyzed to screen out differentially expressed miRNA and differentially expressed genes (DEGs). Next, target genes of differentially expressed miRNA were predicted, and differentially expressed miRNA-DEG regulatory network was constructed to select key miRNAs. After gene ontology and kyoto encyclopedia of genes and genomes enrichment analysis on target genes of key miRNAs, the target genes enriched in pathways were extracted to establish differentially expressed miRNA-DEG-disease gene network for gene interaction analysis. Targetting relationship between miRNAs and target genes was verified. A total of 15 miRNAs were differentially expressed in NEC in newborn, amongst which miR-429/200a/b and miR-141/200c clusters were poorly expressed and might play a significant role in NEC in newborn. Besides, target genes of miR-429/200a/b and miR-141/200c clusters were enriched in 11 signaling pathways. Vascular endothelial growth factor (VEGFA), E-selectin (SELE), kinase insert domain receptor (KDR), fms-related tyrosine kinase 1 (FLT1), and hepatocyte growth factor (HGF) were highly expressed in NEC in newborn, which were negatively regulated by miR-429/200a/b and miR-141/200c clusters and shared close association with disease genes. miR-429/200a/b and miR-141/200c clusters are poorly expressed while their target genes (VEGFA, SELE, KDR, FLT1, and HGF) are highly expressed in NEC in newborn, which might be identified as important biomarkers for this disease.

## Introduction

Necrotizing enterocolitis (NEC) is a devastating disease amongst preterm infants, which is accompanied by chronic neurodevelopmental morbidity and a high death rate ranging from 15 to 30% [1]. Nearly one in 15 infants born 32 weeks before gestation are diagnosed as NEC, amongst which one third face the risk of death [2]. NEC is pathologically characterized by the deterioration of systemic sepsis and the continuous infiltration of a variety of inflammatory cell into the intestinal mucosa of newborns [3]. During the past few decades, the standard treatments for NEC in newborn include measurement of blood pressure, ventilation, parenteral nutrition, broad-spectrum antibiotics, bowel rest, necrotic bowel resection, or peritoneal drainage in aggravated cases [4]. However, those newborns who narrowly survive from NEC still endure a higher morbidity of neurodevelopmental impairment, strictures, and short gut syndrome [5]. Recently, differentially expressed miRNAs have been detected in mouse infected with either ulcerative colitis or ulcerative colitis related colorectal cancer, which might be associated with the initiation, invasion, and migration of colorectal cancer [6]. Yet there are limited data regarding the role and related molecular mechanism of miRNAs in NEC in newborn. Therefore, a comprehensive meta-analysis of miRNAs as novel biomarkers in NEC development is of great significance.

miRNAs play regulatory roles in various biological processes (BPs) and their abnormal expression has been found in a variety of human diseases [7]. More importantly, differentially expressed miRNAs have been identified as potential biomarkers for prognosis and diagnosis of human disease [8]. The miR-141/200c cluster and the miR-429/200a/b cluster are critical members of the miR-200 family, which are gathered at two locations in the genome [9]. Recently, several miRNAs including miR-1290 and miR-431 have been reported to be associated with the diagnosis and treatment of NEC [10,11]. Vascular endothelial growth factor (VEGFA) and its receptors 1 fms-related tyrosine kinase 1 (FLT1) and kinase insert domain receptor (KDR) have been newly recognized as critical regulators of angiogenesis and inflammation in colorectal cancer [12]. Dysregulated intestinal VEGF level has been detected in experimental and human NEC, which might be related with the pathogenesis of NEC [13]. E-selectin (SELE) is a prominent regulator of leukocyte-endothelial adhesion, whose high intestinal level are correlated with poor prognosis of NEC [14]. Hepatocyte growth factor (HGF) is a paracrine hormone that is vital for morphology, motility, and proliferation of epithelial cells [15]. HGF delivered by amniotic fluid exerts a protective effect on experimental rat pups with NEC [16]. Given the former analyses, the present study was expected to make contributions to identify novel biomarkers for therapy of NEC in newborn. Hence, the present study analyzed NEC miRNA and gene expression chips, and then constructed differentially expressed miRNA differentially expressed genes (DEGs) regulatory network to recognize potential miRNAs and genes in NEC therapy.

## Materials and methods

### Data sources

NEC in newborn-related microarray data were downloaded from Gene Expression Omnibus (GEO) database (https://www.ncbi.nlm.nih.gov/geo/), the sub-database of National Center for Biotechnology Information, amongst which miRNA chip GSE68054 and gene chip GSE46619 were utilized for screening differential expression. GSE68054 and GSE46619 included expression data of intestinal tissues from newborns with NEC and spontaneous enterobrosis. Next, miRNA expression data of four newborns with NEC and four control samples were obtained from GSE68054 for differential analysis and screening of differentially expressed miRNA from NEC newborns. Then, gene expression data of five newborns with NEC and four control samples were obtained from GSE46619 to screen out DEG.

### Differential analysis of expression data

After expression data and gene annotation files of GSE68054 and GSE46619 were downloaded from GEO database, differential analysis was conducted by R language in order to screen out DEG or differentially expressed miRNA. Affy package of R language was used for standardized pretreatment [17], while limma package was applied for screening of DEG or differentially expressed miRNA [18]. After correction, the *P* value was expressed as *adj.p.Val* and log2-fold change (log2FC) value of miRNA or gene was calculated, where |log2FC| >1 and *adj.p.Val* <0.05 were considered as differentially expressed. The volcano map of differential expression was plotted to monitor the distribution of DEG, and pheatmap package of R language was used to draw heat map.

### Differentially expressed miRNA-DEG regulatory network construction

DIANA TOOLS database (http://diana.imis.athena-innovation.gr/DianaTools/index.php?r=microT_CDS/index) is based on high throughput immunoprecipitation and sequencing data from mammals, which can be used to predict target genes of miRNA [19]. After the screening of differentially expressed miRNA from miRNA chip GSE68054, the target gene of these miRNAs was predicted. Next, Cytoscape 3.6.0 software was utilized to construct visualized differentially expressed miRNA-DEG network based on the prediction results of differentially expressed miRNA from GSE68054 and DEG from GSE46619, from which the key miRNAs were selected [20]. In addition, TargetScan (http://www.targetscan.org/vert_71/), a prediction website for miRNA target gene, was used to predict the target gene through conservative 8mer, 7mer, and 6mer sites, and provide target binding sites. TargetScan was further applied in subsequent verification of the potential relationship between differentially expressed miRNA and DEG [21].

### Gene ontology and kyoto encyclopedia of genes and genomes enrichment analysis

After the key miRNA was selected from constructed differentially expressed miRNA-DEG regulatory network, the regulatory DEG of miRNA was extracted to conduct functional analysis in order to further explore the potential effects of miRNA. ClusterProfiler package of R language (http://www.bioconductor.org/packages/3.5/bioc/html/clusterProfiler.html) was employed for gene ontology (GO) enrichment analysis. Benjamini-Hochberg method was wielded for multiple hypothesis correction, and *P*<0.05 was regarded as significantly enriched. ClueGO is a plug-in of Cytoscape that can carry out KEGG enrichment analysis and graphically present the kyoto encyclopedia of genes and genomes (KEGG) enrichment results as well as the relationship between gene and pathway [22]. Hence, it was used in KEGG enrichment analysis of target gene of miRNA.

### Differentially expressed miRNA-DEG-disease gene network construction

MalaCards (https://www.malacards.org/) is a database that provides various clinical and genetic annotation information of human diseases, and can be used to retrieve disease-related gene set [23,24]. NEC in newborn-related genes was retrieved in MalaCards with ‘NEC in fetus or newborn’ as keyword. DiGSeE (http://210.107.182.61/geneSearch/) is a search engine of disease gene that obtains evidence from MEDLINE abstract and excavates gene-disease relations [25]. Disease-related genes were retrieved using DiGSeE, with ‘NEC in fetus or newborn’ as keyword. Next, jvenn (http://jvenn.toulouse.inra.fr/app/example.html) was employed to compare the results of disease-related genes from the above two database. Venn map was plotted, and the intersection was obtained as NEC in newborn-related genes for subsequent analysis. Disease-related genes and DEGs were included into String database (https://string-db.org/) to analyze gene–gene interaction. NEC differentially expressed miRNA-DEG-disease gene network was constructed on the basis of differentially expressed miRNA-DEG relations and then visualized in Cytoscape.

## Results

### Differentially expressed miRNA analysis of NEC in newborn

**Figure 1:**
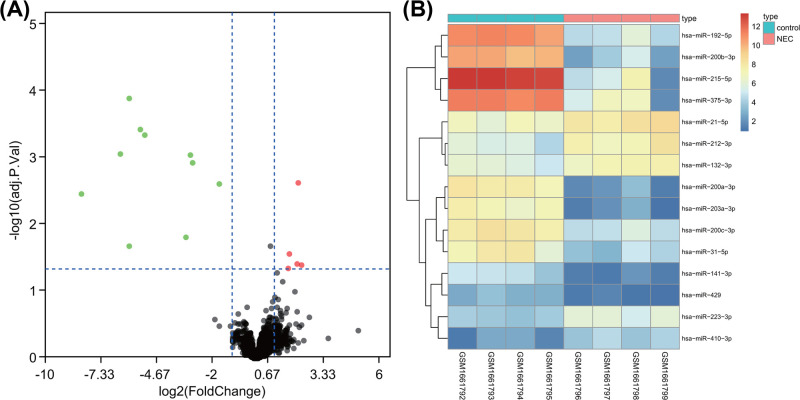
Differential analysis on miRNA chip GSE68054 (**A**) Volcano map of differentially expressed miRNA in miRNA chip GSE68054 of NEC in newborn. Green dot represents down-regulated miRNA and red dot represents up-regulated miRNA in NEC in newborn; gray dot represents normally expressed miRNA. (**B**) Expression heat map of 15 differentially expressed miRNAs in miRNA chip GSE68054 of NEC in newborn. Abscissa stands for sample number, and ordinate stands for differentially expressed miRNA; each rectangle represents the expression of each sample.

R language was utilized to analyze expression data of NEC in newborn. Primarily, 15 differentially expressed miRNAs were selected from miRNA chip GSE68054 according to |logFC| >1 and *adj.p.Val* <0.05. As depicted in Figure 1A,B, five miRNAs (hsa-miR-223-3p, hsa-miR-21-5p, hsa-miR-410-3p, hsa-miR-212-3p, and hsa-miR-132-3p) were highly expressed, while other ten miRNAs (hsa-miR-192-5p, hsa-miR-200a-3p, hsa-miR-203a-3p, hsa-miR-200b-3p, hsa-miR-200c-3p, hsa-miR-141-3p, hsa-miR-429, hsa-miR-215-5p, hsa-miR-31-5p, and hsa-miR-375-3p) were poorly expressed in newborn with NEC.

### DEG analysis of NEC in newborn

**Figure 2:**
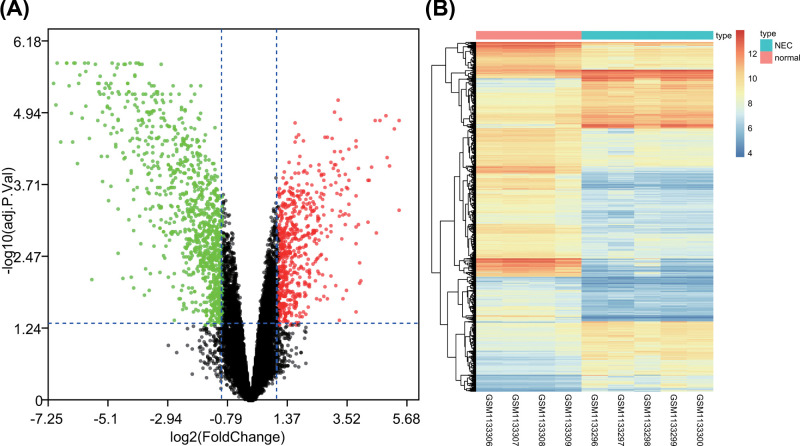
Differential analysis on gene chip GSE46619 (**A**) volcano map of DEG in gene chip GSE46619 of NEC in newborn. Green dot represents down-regulated gene and red dot represents up-regulated gene in NEC in newborn; gray dot represents normally expressed gene. (**B**) Expression heat map of DEGs in gene chip GSE46619 of NEC in newborn. Abscissa stands for sample number, and ordinate stands for DEG; each rectangle represents the expression of each sample.

Next, differential analysis was performed on gene chip GSE46619 of NEC in newborn, and 1538 DEGs were found in NEC in newborn according to |logFC| >1 and *adj.p.Val* <0.05 (Figure 2A,B). The expression of 567 genes was higher, while that of other 971 genes was lower in NEC in newborn than in the control subjects.

### miR-429/200a/b and miR-141/200c clusters might play an important role in NEC in newborn

**Figure 3:**
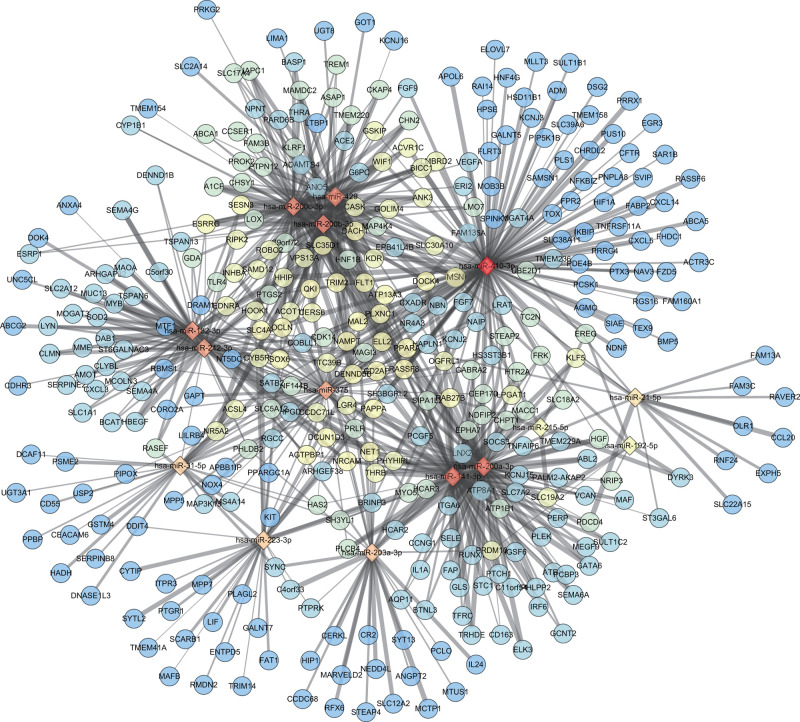
Differentially expressed miRNA-DEG regulatory network Rhombus represents differentially expressed miRNA in NEC in newborn, and circle represents DEG in NEC in newborn; red stands for more target relations, while blue stands for fewer target relations; the bolder edge represents the higher miTG score of differentially expressed miRNA-DEG.

Afterward, the target genes of 15 differentially expressed miRNAs from GSE68054 were predicted using DIANA TOOLS database according to miTG score >0.8. Then, the relationship between DEG and differentially expressed miRNA in GSE46619 was analyzed to construct differentially expressed miRNA-DEG target regulatory network (Figure 3). The number of DEGs that were regulated by differentially expressed miRNA is shown in Table 1, amongst which hsa-miR-410-3p had the most target gene number. Hsa-miR-429, hsa-miR-200b-3p, and hsa-miR-200c-3p gathered as they had more common target genes, so were hsa-miR-141-3p and hsa-miR-200a-3p. These five miRNAs belong to two miRNA clusters. Hsa-miR-429, hsa-miR-200a-3p, and hsa-miR-200b-3p were classified into one cluster, and hsa-miR-141-3p and hsa-miR-200c-3p were categorized into another, which were considered as the key miRNAs to exert significant effects on NEC in newborn.

**Table 1:** The number of DEG regulated by differentially expressed miRNA

miRNA ID	Number	up-/down-regulation
hsa-miR-410-3p	130	Up
hsa-miR-141-3p	82	Down
hsa-miR-200a-3p	82	Down
hsa-miR-200b-3p	72	Down
hsa-miR-429	70	Down
hsa-miR-200c-3p	69	Down
hsa-miR-132-3p	60	Up
hsa-miR-212-3p	54	Up
hsa-miR-375	49	Down
hsa-miR-203a-3p	34	Down
hsa-miR-223-3p	30	Up
hsa-miR-31-5p	25	Down
hsa-miR-21-5p	16	Up

Num. represents the number of DEG regulated by differentially expressed miRNA.

### GO enrichment regarding target genes of miR-429/200a/b and miR-141/200c clusters

**Figure 4:**
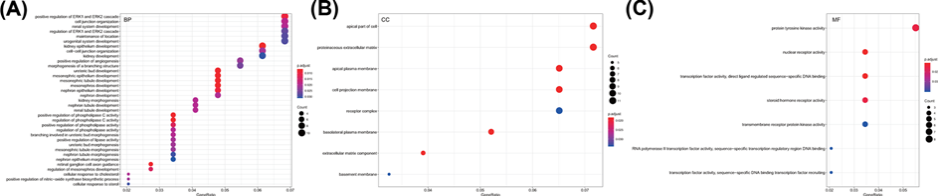
GO enrichment analysis of miR-429/200a/b and miR-141/200c clusters target genes (**A**–**C**) results of enriched miR-429/200a/b and miR-141/200c clusters target genes in BP, CC, and MF, respectively. Ordinate represents specific contents of GO; the larger circle stands for more gene number in this part; the color of circle represents *P* value; the redder color stands for the smaller *P* value.

After analysis on differentially expressed miRNA-DEG regulatory network, the regulatory DEGs of miR-429/200a/b and miR-141/200c clusters were extracted for GO enrichment analysis by R language (Figure 4). GO analysis included three independent ontologies, BP, molecular function (MF), and cellular component (CC), which was performed gene annotation and functional enrichment analysis through gene classification on the basis of different functions. DEGs were enriched in BP (kidney epithelium development, positive regulation of phospholipase C activity, regulation of phospholipase C activity, ureteric bud development, mesonephric epithelium development, etc.) (Figure 4A), CC (apical plasma membrane, apical part of cell, proteinaceous extracellular matrix, basolateral plasma membrane, cell projection membrane, etc.) (Figure 4B), and MF (nuclear receptor activity, transcription factor activity, direct ligand regulated sequence-specific DNA binding, steroid hormone receptor activity, protein tyrosine kinase activity, RNA polymerase II transcription factor activity, sequence-specific transcription regulatory region DNA binding) (Figure 4C).

### KFGG enrichment of target genes of miR-429/200a/b and miR-141/200c clusters

**Figure 5:**
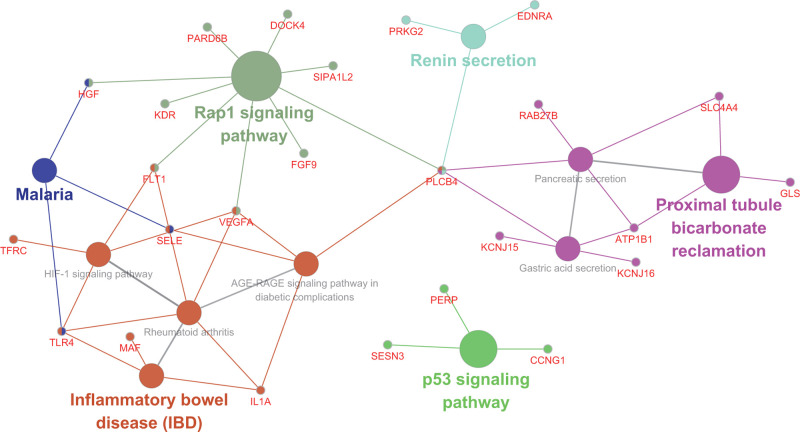
KEGG enrichment analysis of miR-429/200a/b and miR-141/200c clusters target genes The smallest circle represents genes, and the larger circle represents related signaling pathways enriched by genes.

In order to analyze the regulatory mechanism that target genes might be involved in, targetted DEGs of miR-429/200a/b and miR-141/200c clusters were subjected to the KEGG enrichment analysis by Cytoscape plug-in ClueGO, and disclosure of correlation between genes and enriched pathways. As described in Figure 5, target genes were significantly enriched in repressor activator protein 1 (Rap1), HIF-1, p53, renin secretion, proximal tubule bicarbonate reclamation, gastric acid secretion, pancreatic secretion, malaria, inflammatory bowel disease, and rheumatoid arthritis signaling pathways as well as advanced glycation end-product (AGE)-receptor for advanced glycation end-product (RAGE) signaling pathway in diabetic complications. Recent studies have demonstrated that HIF-1, Rap1, AGE (RAGE), and p53 were critical for multiple colitis [26–29]. Genes related with the above signaling pathways were regulated by miR-429/200a/b and miR-141/200c clusters, which were applied in subsequent analysis to investigate their association with NEC in newborn.

### Analysis of differentially expressed miRNA-DEG disease gene network

**Figure 6:**
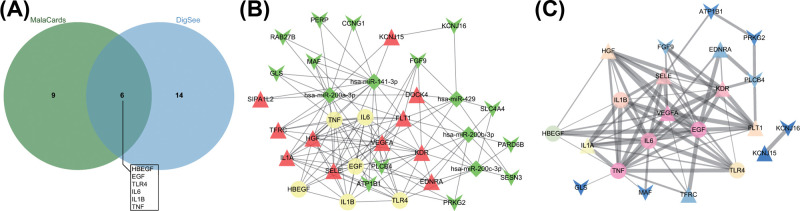
Differentially expressed miRNA-DEG-disease gene network of NEC in newborn (**A**) comparison of disease genes from MalaCards and DiGSeE finds six intersection genes. (**B**) Differentially expressed miRNA-DEG-disease gene interaction network. Yellow circle represents disease gene; red triangle represents up-regulated DEG; green arrow represents down-regulated DEG; rhombus represents down-regulated differentially expressed miRNA. (**C**) Subnetwork of DEG-disease gene interaction. Arrow represents down-regulated DEG; triangle represents up-regulated DEG; the bolder edge stands for the larger combined score amongst genes; genes presented as pink stands for higher association with other genes.

MalaCards and DiGSeE were used to retrieve NEC newborn-related genes. In total, six intersection genes (HBEGF, EGF, TLR4, IL6, IL1B, and TNF) were found according to the results of comparison between 15 predicted disease genes from MalaCards and the top 20 genes from DiGSeE showed, which were utilized in following analysis as disease genes (Figure 6A). Next, string database was employed to analyze the interaction between DEGs and disease genes, and construct differentially expressed miRNA-DEG disease gene interaction network on the basis of differentially expressed miRNA-DEG relation (Figure 6B). The results showed that miR-429/200a/b and miR-141/200c clusters were related to disease genes by regulating a variety of target genes. Molecules with higher relevancy with other molecules (degree ≥10) were regarded as hub molecule, and target genes KDR, VEGFA, FLT1, SELE, and HGF were differentially expressed and regulated by miR-429/200a/b and miR-141/200c clusters (Table 2). Their effects on NEC in newborn might be linked to disease genes. In order to clearly present the interaction between target genes and disease genes, subnetwork was extracted (Figure 6C), which demonstrated VEGFA, KDR, and SELE as closely associated DEGs.

**Table 2:** Hub molecule of differentially expressed miRNA-DEG-disease gene network in NEC in newborn

Name	Type	Degree	Up-/down-regulation
hsa-miR-141-3p	Differentially expressed miRNA	15	Down
hsa-miR-200a-3p	Differentially expressed miRNA	15	Down
KDR	DEG	15	Up
VEGFA	DEG	15	Up
TNF	Disease gene	14	-
EGF	Disease gene	14	-
FLT1	DEG	14	Up
SELE	DEG	13	Up
IL6	Disease gene	13	-
HGF	DEG	11	Up
TLR4	Disease gene	10	-
IL1B	Disease gene	10	-

### Abnormally expressed miR-429/200a/b and miR-141/200c clusters might affect the progression of NEC in newborn by regulating VEGFA, SELE, KDR, FLT1, and HGF

**Figure 7:**
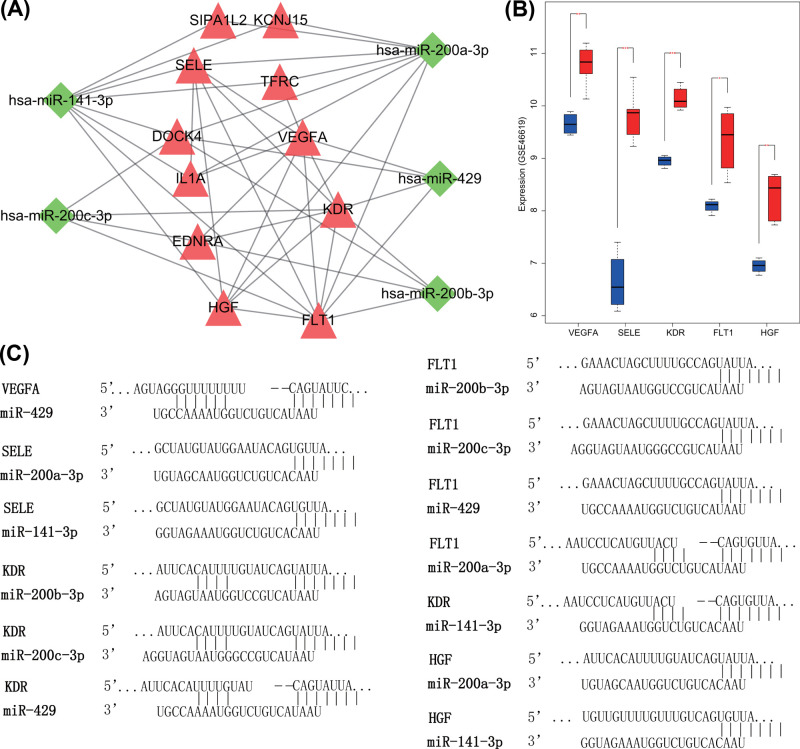
miR-429/200a/b and miR-141/200c clusters play a role in NEC in newborn progression by targetting VEGFA, SELE, KDR, FLT1, and HGF (**A**) The interaction amongst miR-429/200a/b, miR-141/200c clusters, and their up-regulated target genes. (**B**) Expression of VEGFA, SELE, KDR, FLT1, and HGF in gene chip GSE46619 of NEC in newborn. (**C**) Target binding sites between miR-429/200a/b or miR-141/200c clusters and VEGFA, SELE, KDR, FLT1, or HGF predicted by TargetScan.

miRNAs have been generally considered to negatively regulate its target genes. Both miR-429/200a/b and miR-141/200c clusters were poorly expressed in NEC in newborn. Hence, up-regulated target genes in NEC in newborn were extracted (Figure 7A). Five hub DEGs (VEGFA, SELE, KDR, FLT1, and HGF) selected from differentially expressed miRNA-DEG-disease gene network all revealed to be highly expressed in NEC in newborn (Figure 7B). In addition, the potential relationship between hub DEGs and miR-429/200a/b and miR-141/200c clusters was verified in TargetScan, and their binding sites are shown in Figure 7C. Hub DEGs might be regulated by single or multiple miRNAs, and the interaction amongst DEGs and disease genes was tight, which might be viewed as important biomarkers for NEC in newborn. For example, VEGFA overexpression was related with ulcerative colitis [30] and various signaling pathways according to KEGG enrichment results (Figure 5). In addition, KDR and FLT1 were two receptors of VEGFA, which were differentially expressed in NEC in newborn. Moreover, VEGFA could interact with its receptors (KDR and FLT1), DEGs (SELE, HGF, IL1A, etc.) and disease genes. Therefore, the aberrant expression of miR-429/200a/b and miR-141/200c clusters in NEC in newborn might influence its development via regulation of VEGFA, SELE, KDR, FLT1, and HGF.

## Discussion

NEC is a severe disease mainly induced by improper bacterial colonization with enterobacteriaceae, which affects about 10% of preterm infants with an extremely high mortality [2]. Recently, miRNAs have been reported to be associated with disease pathophysiology and mucosa integrity of NEC as well as spontaneous intestinal perforation [31]. Collectively, based on analysis of NEC miRNA and gene expression chips and the construction of differentially expressed miRNA-DEG-disease gene regulatory network, our study provided evidence that miR-429/200a/b and miR-141/200c clusters might serve as important biomarkers for NEC in newborn by binding to VEGFA, SELE, KDR, FLT1, and HGF.

Initially, according to differential analysis on NEC miRNA expression chip, miR-429/200a/b and miR-141/200c clusters all displayed poor expression in NEC in newborn. miR-429 has been identified as a candidate for anticolitis treatment in ulcerative colitis, whose down-regulation has been determined in colitis induced by dextran sulphate sodium [32]. Besides, a recent study has revealed that miR-200a exhibits decreased expression in inflamed colonic region of ulcerative colitis patients [33]. In addition, miR-200b is poorly expressed in the intestinal epithelial cell-6 and colon, and its up-regulation by miR-200b-containing microvesicle alleviates intestinal fibrosis-associated experimental colitis [34]. Additionally, poor expression of miR-200c-3p has been detected in ulcerative colitis [35]. Moreover, expression of miR-141 is shown to be significantly lower in patients with active ulcerative colitis [36]. miR-141 overexpression contributes to alleviated experimental colitis in the IL-10 knockout or 2,4,6-trinitrobenzene sulfonic acid-induced chronic colitis mice [37].

Besides, differential analysis on NEC gene expression chip found that VEGFA, FLT1, KDR, SELE, and HGF expressed highly in NEC in newborn. VEGF therapy has been newly proved to be a promising treatment method for ischemic bowel diseases such as NEC [38]. VEGFA is known as a strong vascular and angiogenic permeability factor, which promotes endothelial cell permeability, migration, and growth [39]. A prior study has revealed that overexpressed VEGFA in ulcerative colitis accelerates inflammatory angiogenesis of the colon, thus promoting the progression of ulcerative colitis [30]. VEGFA interacts with its receptors FLT1 and KDR has been suggested to play a regulatory role in progressive angiogenesis by triggering its downstream signaling pathway [40]. KDR serves as a predominant regulator of angiogenesis and mitosis, which is considered to be involved in endothelial survival and permeability [41]. Both VEGFA and KDR exhibit at a high level in the intestine before birth in a neonatal mouse NEC model [42]. FLT1, a tyrosine kinase receptor, acts as a promoter in cancer growth and metastasis, whose affinity with VEGFA is approximately ten times higher than KDR [43]. FLT1 has been proved to be highly expressed in nodal metastases and primary tumors of colorectal cancer, whose overexpression leads to local recurrence and lymphovascular invasion [44]. SELE is a surface marker of endothelial cell inflammation, which stimulates intriguing docking sites for drug delivery mediated by inflamed endothelium and the monocyte adherence to endothelial cells [45]. SELE is up-regulated on activated endothelium, and elevated intestinal SELE level leads to a poor outcome and multiple organ failure in newborns with NEC [14]. HGF is secreted by mesenchymal and stromal cells, which plays a significant role in epithelial cellular processes [46]. HGF is abundantly enriched in amniotic fluid of rat pups with NEC, whose interruption is associated with progression of NEC in newborn [16]. All in all, these findings proved our result that VEGFA, FLT1, KDR, SELE, and HGF were highly expressed in NEC in newborn.

Additionally, GO and KEGG enrichment analysis showed that miR-429/200a/b and miR-141/200c clusters might affect NEC progression by regulating VEGFA, FLT1, KDR, SELE, and HGF. A former study has demonstrated the negative regulation of the miR-200 family on transcription factors ZEB proteins including twist, slug, snail, ZEB1, and ZEB2, which are implicated in EMT process [47]. Besides, endometrial miR-200c affects cellular angiogenesis, inflammation, and transformation partly through regulating VEGFA and FLT1 [48]. Choi *et al.* has demonstrated that miR-200b exerts its inhibitory effect on angiogenesis through regulation of VEGF, FLT1, and KDR [49]. miR-31 induces the inhibition of colon cancer cell migration and metastasis by suppressing SELE [50]. miR-7-5p contributes to depleted oncogenes in the MCF-10A mammary epithelial cell by regulating HGF activity [51]. Therefore, miR-429/200a/b and miR-141/200c clusters might influence the progression of NEC through regulation of VEGFA, FLT1, KDR, SELE, and HGF.

Taken together, our study demonstrates that miR-429/200a/b and miR-141/200c clusters are identified as potential biomarkers for NEC through regulation of VEGFA, SELE, KDR, FLT1, and HGF, which yields a better understanding for NEC treatment. However, the clinical value of the present study remains to be tapped and verified. Therefore, the clinical efficacy of miR-429/200a/b and miR-141/200c clusters will need to be assessed in current and future clinical trials.

## Abbreviations

AGE: advanced glycation end-product
BP: biological process
CC: cellular component
DEG: differentially expressed gene
FLT1: fms-related tyrosine kinase 1
GEO: Gene Expression Omnibus
HGF: hepatocyte growth factor
KDR: kinase insert domain receptor
MF: molecular function
NEC: necrotizing enterocolitis
RAGE: receptor for AGE
Rap1: Ras-associated protein 1
SELE: selectin E
VEGFA: vascular endothelial growth factor A
